# Reduced glucocorticoid receptor expression in blood mononuclear cells of patients with borderline personality disorder

**DOI:** 10.3389/fpsyt.2022.951373

**Published:** 2022-08-02

**Authors:** José Manuel López-Villatoro, Karina S. MacDowell, Marina Diaz-Marsá, Alejandro De La Torre-Luque, Clara Prittwitz, Alejandra Galvez-Merlin, Juan C. Leza, Jose L. Carrasco

**Affiliations:** ^1^Health Research Institute, Hospital Clínico San Carlos (IdISSC), Madrid, Spain; ^2^Department of Pharmacology and Toxicology, Faculty of Medicine, Universidad Complutense de Madrid (UCM), Madrid, Spain; ^3^Institute of Health Research Hospital 12 de Octubre (imas12), Madrid, Spain; ^4^University Institute of Research in Neurochemistry UCM, Madrid, Spain; ^5^Biomedical Research Networking Consortium for Mental Health (CIBERSAM), Madrid, Spain; ^6^Department of Legal Medicine, Psychiatry and Pathology, Faculty of Medicine, Universidad Complutense de Madrid (UCM), Madrid, Spain; ^7^Faculty of Psychology, Universidad Complutense de Madrid (UCM), Madrid, Spain

**Keywords:** borderline personality disorder, stress, cortisol, glucocorticoid receptor, trauma

## Abstract

**Introduction:**

Abnormal cortisol suppression in borderline personality disorder has been consistently reported in previous studies, suggesting that a hypersensitivity response of the hypothalamic-pituitary-adrenal (HPA) axis might occur in these patients. In this study, the abnormalities of the cortisol response in borderline personality disorder (BPD) are investigated through the cellular expression of the glucocorticoid receptors (GR) in BPD patients and its relationship with traumatic experiences.

**Methodology:**

Sixty-nine male and female patients diagnosed with BPD and 62 healthy controls were studied. Peripheral blood mononuclear cells were obtained to investigate the expression of glucocorticoid receptors. Western blot was used to measure protein expression. Statistical correlations of GR expression with BPD clinical features and intensity of previous traumatic events were investigated.

**Results:**

A significant decrease in the nuclear expression of glucocorticoid receptors was found in BPD patients compared to healthy controls in a regression analysis controlling for the effect of medication. GR expression decrease correlated significantly with clinical levels of anxiety and depression, but not with previous traumatic experiences in patients.

**Conclusions:**

BPD patients had a lower nuclear expression of glucocorticoid receptors than healthy controls, when it was controlled for the effect of medication. The reduced GR expression in BPD patients was not associated with previous traumatic events and might be associated with other aspects of BPD, such as emotional instability; more studies with larger samples of patients are still needed to understand the relevance and the implications of these findings.

## Introduction

Borderline personality disorder (BPD) is a severe and persistent mental disorder characterized by affective instability and impulsive behaviors, affecting self-image and interpersonal relationships (1).

Many studies have proposed that susceptibility and stress reactivity in BPD mediate both the development and maintenance of disorder symptomatology (2). Alterations in some neurotransmitters and neuropeptides related to stress response have been reported in research studies with BPD studies (3, 4). Among them, dysfunctional serotonergic regulation (5) and overactivity of the hypothalamic-pituitary-adrenal (HPA) axis (6) are the most consistent findings.

According to research evidence, one pathogenic factor of abnormal response to stress in borderline personality disorder might be the early experience of traumatic situations in childhood (7). Different studies have described frequent comorbidity between post-traumatic stress disorder (PTSD) and BPD (8, 9). Furthermore, others reported consistent associations between adult BPD and emotional abuse and different types of abuse in childhood periods (10, 11). Some authors have even correlated the severity of sexual abuse to borderline traits (12) and self-destructive behaviors (13).

Given the growing interest in stress reactivity in BPD, multiple studies assessing HPA axis activity have been carried out. However, results to date are inconsistent, due to the heterogeneity of the disorder, multiple forms of HPA axis activity measures studied and differences in operational definitions of stress and trauma (2). Some studies (14, 15) have shown statistically reduced cortisol levels in BPD patients compared to healthy subjects and to patients with other personality disorders while other studies, such as Inoue et al. (16), demonstrated increased cortisol reactivity in BPD patients compared to control subjects.

Some classic studies on the HPA axis response showed an increased cortisol suppression in the dexamethasone suppression test (DST) in borderline patients with and without post-traumatic clinical features compared with healthy controls (7, 17). While others (18) found abnormal HPA-axis response only in BPD patients with previous traumatic experiences and posttraumatic symptoms. According to these authors, whereas BPD patients with trauma-associated symptoms showed normal or reduced basal cortisol levels with no abnormalities in feedback sensitivity as measured by DST, BPD patients without posttraumatic symptomatology tend to show normal or high baseline plasma cortisol levels and reduced feedback sensitivity (18).

There is no clear explanation of the relationship between cortisol hypersupression, post-traumatic stress and borderline personality disorder to date. Some authors hypothesized either an increased glucocorticoid receptor density or receptor hypersensitivity that could be shared for posttraumatic stress disorder and borderline personality disorder (7), explaining the enhanced cortisol suppression in the DST in these groups.

Glucocorticoids (GC) bind to the glucocorticoid receptor (GR), a member of the nuclear receptor (NR) family of intracellular receptors, which also contains the progesterone receptor (PR), estrogen receptor (ER) androgen receptor (AR), mineralocorticoid receptor (MR), and several orphan receptors (19). GR is inactive in the cytoplasm bound to a complex of chaperones. However, when GC binds to the receptor, GR translocates to the nucleus and attaches to specific DNA sequences, promoting the transcription of a series of elements, including inflammatory factors (20). GR has been identified as the main receptor responsible for the physiological and pharmacological effects of GC. The GR is closely related to the MR, which show some cross-reactivity. So, although MR has a high affinity, the study of GR expression is important because synthetic GCs (such as dexamethasone and methylprednisolone) act almost exclusively on the GR (19).

To date, the main studies on glucocorticoid receptor expression have been performed in patients with major depression (21) and in trauma-exposed subjects with and without post-traumatic stress disorder (22), with both profiles showing a decrease in glucocorticoid receptor expression.

According to this proposal, the present study aims to investigate the association of glucocorticoid receptor (GR) expression and its association with BPD symptoms and with traumatic experiences in patients and healthy subjects, in order to search for a possible endophenotype associated with the consistently reported abnormal cortisol responses in BPD patients.

## Materials and methods

### Participants

The sample studied consisted of 69 male and female patients diagnosed with borderline personality disorder as a primary diagnosis, according to the DSM-V criteria (1) and had to have moderate-severe severity (CGI (clinical global impression) >4) and moderate dysfunctionality (GAF (global activity evaluation scale) <65) to enter the study. Patients were recruited from the Personality Disorders Day Hospital of the Hospital Clínico San Carlos and all of them were taking medication treatment at stable doses from the last 2 months (see Table 1).

**Table 1:** Descriptive statistics according to study groups.

	**Patients**	**Controls**	**Contrast test**	***ES***
Sex (% ref.women)	86.76	92.98	0.71	0.1
Age (years)	29.59 (9.62)	27.39 (8.08)	1.39	−0.25
Qualification level (% higher than secondary education)	53.97	85.71 (12.50)	12.50**	0.33
Marital status (% married)	1.59	7.14	1.1	0.13
Working status			55.79**	0.65
Unemployed	74.6	7.14		
Student	19.05	57.14		
Employed	6.35	35.71		
Antidepressant prescription (% no)	73.02	100	65.26**	0.72
Antipsychotic prescription (% no)	44.44	100	31.09**	0.51
Benzodiazepine prescription (% no)	63.49	100	52.18**	0.65

Percentage of cases are displayed for dichotomous and categorical variables. Means and standard deviation (between brackets) are displayed for continuous variables. The t-based test was used for continuous variable contrast test. The χ^2^-based test was used for dichotomous/categorical variable contrast test. **Contrast tests were significant with p < 0.01. ES = effect size estimate (Cohen's d for continuous outcomes and Cramer's V for non-continuous outcomes).

Patients were excluded from the study if they met the following criteria: (1) had a neurological or medical illness that could affect brain functions; (2) had an IQ <85; (3) had a lifetime history of schizophrenia, schizophreniform disorder or bipolar disorder; (4) had a major depressive episode or a substance use disorder that could affect neuropsychological performance at the time of the study; (5) had a corticoid treatment during the last month.

The sample of control participants consisted of a group of 62 people with similar gender and age distribution to the patients. Controls were healthy with no medical or neurological disease and IQ >85 and were recruited through advertisements in different social settings.

All patients and controls received detailed information about the study and signed written informed consent before participating in the research. The clinical research study was approved by the Clinical Research Ethics Committee of the Hospital Clínico San Carlos.

### Instruments

Experienced psychiatrists and psychologists carried out the collection of clinical variables at the beginning of the study. All patients and controls were interviewed with the Structural Interview for Personality Disorders [SCID-II, (23)]. Severity was measured with the Clinical Global Scale for Personality Disorders [CGI-BPD, (24)] and chronicity was assessed with the Global Assessment of Functioning Scale [GAF, (25)]. The presence of traumatic experiences was measured with the Traumatic Experiences Questionnaire [TQ, (26)]. Other clinical variables, such as anxious and depressive symptoms, were assessed with the Hamilton Anxiety Rating Scale [HARS, (27)] and the Montgomery-Asberg Depression Scale [MADRS, (28)].

### Specimen collection and preparation

Venous blood samples (10 mL) were collected between 8:00 and 10:00 h after fasting overnight. Blood tubes were centrifuged (641g × 10 min, 4°C). The resultant plasma samples were collected and stored at −80°C. The rest of the sample was 1:2 diluted in culture medium (RPMI 1640, LifeTechnologies) and a gradient with Ficoll-Paque (GE Healthcare) was used to isolate mononuclear cells by centrifugation (800g × 40 min, room temperature -RT-). Peripheral Blood Mononuclear Cell (PBMC) layer was aspired, resuspended in RPMI and centrifuged (1116 g × 10 min, RT). The supernatant was removed and the mononuclear cell-enriched pellet was stored at −80°C.

### Biochemical determinations in peripheral blood mononuclear cell (PBMC)

PBMC samples were fractionated in cytosolic and nuclear extracts as described in previously published articles (29, 30). The protein levels from PBMCs nuclear extracts were measured using the Bradford method, based on the protein-dye binding.

Western Blot (WB), 15 μg of nuclear extracts were loaded into an electrophoresis gel. Once separated based on molecular weight, proteins from the gels were blotted onto a nitrocellulose membrane (Transfer Pack, BioRad) with a semi-dry transfer system (Bio-Rad) and were blocked in 5% BSA for 1.5 hr, then the membranes were incubated overnight at 4°C with specific antibodies against glucocorticoid receptor GR (1:1000 BSA1%, sc1003, SCB), as internal control GAPDH (1:5000, G8795, Sigma) and β-actin (A5441, 1:10000 TBSt; Sigma) were used. After washing with a TBS-Tween solution the membranes were incubated with the respective horseradish peroxidase-conjugated secondary antibodies for 90 min at room temperature and revealed by ECLTM-kit following manufacturer's instructions (Amersham Ibérica, Spain). Blots were imaged using an Odyssey^®^ Fc System (Li-COR Biosciences) and quantified by densitometry (NIH ImageJ^®^ software). GAPDH was used as loading control. Results are expressed as arbitrary units of optical density (O.D.) as a percentage compared to CT groups. The WB were repeated at least three times in separate assays, to ensure the reproducibility of the results.

### Data analysis

Descriptive statistics on sociodemographic factors were compared between the study groups using the χ2 test for categorical/binary data and *t*-tests for continuous data. The Cramer's V (categorical data) and the Cohen's d (continuous measures) estimates were calculated as effect size estimates.

Multiple linear regression was conducted to study whether the study groups showed significantly different levels of glucocorticoid receptors (under a loglinear transformation). Sex and age were used as a covariate within the model. A model comparison strategy was followed to select the regression model that explained better the outcome (glucocorticoid receptor level) variance. In this sense, three regression models were estimated: unconstrained (model without covariates), sociodemographic model (model with sex and age covariates) and full model (adding the study group factor). Model selection was based on The Akaike information criterion (AIC) was used for model selection, observing a better fit to data with a lower AIC. The B coefficient (with 95% confidence interval) was used as a loading estimate.

The same rationale (i.e., multiple linear regression) was followed to test the influence of clinical factors (i.e., levels of depressive symptoms, anxiety and trauma experience) on glucocorticoid receptor level in the clinical group.

All the analyses were conducted using the R Software (packages psych and lme4)

## Results

Sociodemographic characteristics and functional measures of patients and controls are shown in Table 1.

**Figure 1:**
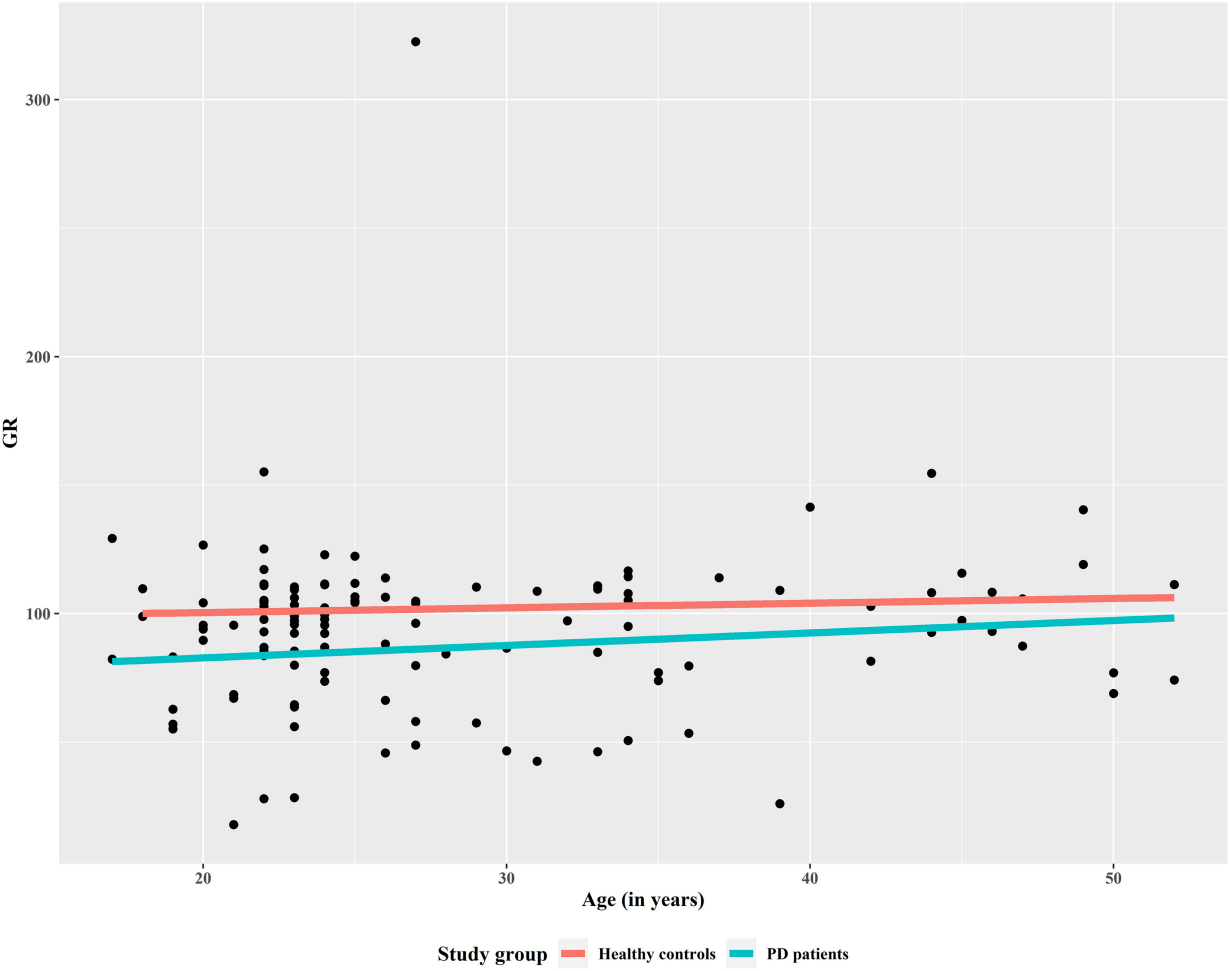
Predictors in the regression analysis to explain glucocorticoid receptor expression, controlled for the antipsychotic prescription. This regression model showed a lower Akaike information criterion (AIC = 79.96) than the unconstrained model (AIC = 83.47) and the model with sociodemographic predictors (AIC = 86.77).

In a first global analysis, no differences were found between medicated BPD patients and healthy control group for nuclear expression of GR (x = 41.46 and x = 15.36, respectively), but significant differences appeared when the regression analysis was controlled for the effects of medication, particularly antipsychotics and mood stabilizers, showing significant differences in GR expression between patients and controls, with BPD patients showing lower GR expression (Figure 1).

The correlations of glucocorticoid receptor expression and traumatic experiences (Traumatic Experiences Questionnaire TQ), depression (Montgomery-Asberg Depression Scale, MADRS) and anxiety (Hamilton Anxiety Scale, HARS) symptomatology, was investigated in BPD patients controlling for the effect of antipsychotic medication (Table 2). The results showed no statistically significant correlation between GR expression and the Traumatic Experiences Questionnaire score (t = 0.71) in borderline patients. However, a statistically significant positive correlation was found between GR expression and MADRS score (t = 2.95), and a statistically significant negative correlation with HARS score (t = −2.15).

**Table 2:** Clinical predictors to explain glucocorticoid receptors in the patient group, controlled for the antipsychotic prescription.

**Variable**	***B***	***SE***	***t***
(Intercept)	4.45	0.05	83.12**
Depression symptoms	0.19	0.06	2.95**
Anxiety	−0.14	0.07	−2.15*
Traumatic experience	0.04	0.05	0.71

The regression model showed lower Akaike information criterion (AIC = 67.09) than the unconstrained model (AIC = 82.25). Depression was measured using the Montgomery-Asberg Depression Rating Scale (MADRS). Anxiety symptoms were measured by means of the Hamilton Anxiety Rating Scale (HARS). Traumatic experiences were assessed using the Trauma Questionnaire (TQ). B = Factor loading. SE = Standard error of the loading. *Contrast tests were significant with p < 0.05. **Contrast tests were significant with p < 0.01.

## Discussion

This study indicates that abnormal functioning of glucocorticoid receptors at the cellular level might be found in borderline personality disorder, which is concordant with a vast amount of previous literature on the relationship between Hypothalamic-pituitary-adrenal axis (HPA) dysfunctions and BPD.

Stress-related ACTH released is physiologically regulated by circulating glucocorticoids through negative feedback mechanisms at glucocorticoid receptors (GR) in the pituitary, the hypothalamus and the hippocampus. This negative feedback loop is critical for the modulation of the stress response and largely depends on the normal functioning of GR at the pituitary and other areas (18). A low cortisol suppression in the dexamethasone test suggests a reduced sensitivity of GR while the enhanced cortisol suppression after dexamethasone administration indicates GR hypersensitivity (7).

Our results show that GR expression in borderline patients is significantly lower than in healthy controls, when it was controlled for the effects of medication, particularly antipsychotics and mood stabilizers. In the clinical practice, antipsychotic drug treatment in BPD patients with high severity is used for its ability to stabilize mood. According to the results of our study, the stabilizing effect of these drugs could lie in the activation of GR receptors as an anti-inflammatory pathway.

These results could suggest a deficient activation pathway in patients with BPD under basal conditions. However, the levels of expression studied here correspond to the nuclear fraction, that is, receptors that have been activated by the union of GC. This leaves a large amount of inactive GR in the cytoplasm waiting to be activated in response to any potential stimulus. Previous studies on the HPA axis (7) suggested altered feedback inhibition at the HPA axis by using a very low dose (0,25 mg) dexamethasone test that induced a significantly greater cortisol suppression in BPD patients compared with healthy controls. The GR expression levels here exposed, could be immediately interpreted as contrary to the hypersensitivity hypothesis. However, these levels do not reflect how the HPA axis will be activated in this group of patients when stimulated, the concept of receptor nuclear expression is not equivalent to receptor sensitivity and decreasing receptor protein synthesis might act as a downregulation mechanism of a previous receptor oversensitivity. On the other hand, the results are concordant with previous studies showing a reduced suppression of cortisol after DST (18), which could be associated with reduced levels of GR as found in our study.

Also, our study investigated the relationship between GR expression and clinical symptomatology in BPD patients controlling for the effect of antipsychotic medication. Our BPD patients showed significantly greater scores in the traumatic scales than healthy controls, although few of them presented typical post-traumatic symptoms. However, our results do not show a statistically significant relationship between GR expression and the Traumatic Experiences Questionnaire (TQ) score in these patients. In contrast, we observed a statistically significant positive correlation between GR expression and the MADRS Depression scale score and also a statistically significant negative correlation between GR expression and the HARS Anxiety scale score, with BPD patients presenting higher depressive symptoms and lower anxiety showing greater expression of glucocorticoid receptors.

These results are contrary to those expected according to the studies of Wingenfeld et al. (18) and Carrasco et al. (7), who found alterations in the HPA axis response in relation to trauma-associated symptoms in BPD. Divergence with the expected results could be due to the nature of the construct of the Traumatic Experiences Questionnaire TQ. This questionnaire assesses lived experiences more related to post-traumatic stress disorder (PTSD) than to complex trauma, such as attachment trauma. Sabo (31) already reported the influence of attachment trauma, such as biparental neglect or overprotection, on the development of BPD. During childhood and adolescence, apathetic or irregular affective responses from caregivers could help the patients learn to cope with stress through inappropriate coping methods (32), producing a continuous activation of the stress system and permanent abnormalities in the HPA axis. Therefore, the alterations observed in the HPA axis response in BPD patients could be due more to the continuous and frequent experiences of complex trauma than to punctual exposures to significant events, which is more associated with simple trauma and post-traumatic stress disorder.

In addition, complex trauma associated with emotional neglect or overprotection, contribute to the development of distorted views of oneself and others (33), producing significant emotional instability. Our results show a relationship between glucocorticoid receptor expression and symptoms of emotional instability, such as depression and anxiety, controlling the effects of medication, which could be due to an underlying complex trauma. However, in order to confirm this hypothesis, future studies should collect in detail the exposure to complex trauma experiences in BPD patients, through clinical tests more appropriate to this phenomenon.

GR low expression in the BPD group might be part of the global inflammatory activation described in previous studies on BPD (29, 34), who described increased inflammatory factors in borderline patients compared to controls. Furthermore, this inflammatory increase has a compensatory mechanism of anti-inflammatory negative feedback inhibition of the HPA axis García-Bueno et al. (35). In this way, the lower expression of GR in blood white cells could be the consequence of the increase of the pro-inflammatory cytokines and the intracellular inflammatory factors found in BPD patients (34).

To overcome the limitation imposed by medication use in these patients, the analysis of GR expression differences was controlled for the effect of drugs. In fact, differences in GR expression between groups were not statistically significant previous to the analysis controlling for antipsychotic medication, suggesting that these drugs might have specific effects at some stage of the GR expression process. Further analysis controlling for the intake of antidepressants or benzodiazepines in our study did not change the global results. As mentioned above, our sample of BPD patients included severely dysfunctional subjects at a day hospital intensive therapy. Consequently, a large group of them (44%) were receiving treatment with second-generation antipsychotics to achieve affective and behavioral stabilization (Table 1).

Another limitation of the study is the sample size, due to the complexity of the collection of biological samples in this study, which prevents an adequate statistical analysis of the relationship between glucocorticoid receptor expression and the Traumatic Experiences Questionnaire TQ score.

A final limitation derives from the severity of BPD patients in our sample recruited in a day hospital, with a clinical severity at the CGI scale greater than 4 and dysfunctionality lower than 65 at the GAF. Therefore, the findings of our study could probably not be representative of all BPD patients probably do not represent the average BPD population.

## Conclusions

The results of the study highlight the alteration of the hypothalamic-pituitary-adrenal (HPA) axis in patients with borderline personality disorder, showing a lower expression of glucocorticoid receptors in these patients compared to control subjects, being the first to study the expression of glucocorticoid receptors (GR) in patients with BPD. These results are only observed when controlling for the effect of antipsychotic drugs in BPD patients, highlighting the influence of antipsychotics on HPA axis activity. However, this alteration in GR expression in BPD patients is not related to post-traumatic stress symptomatology, suggesting that it would be interesting to extend the clinical investigation of HPA axis alteration in these patients to complex trauma experiences, such as parental neglect or overprotection that are not typically associated with posttraumatic stress disorder symptoms.

## Data availability statement

The original contributions presented in the study are included in the article/supplementary material, further inquiries can be directed to the corresponding author.

## Ethics statement

The studies involving human participants were reviewed and approved by Clinical Research Ethics Committee of the San Carlos Clinical Hospital. The patients/participants provided their written informed consent to participate in this study.

## Author contributions

JL-V, KM, MD-M, AD, CP, and AG-M: critical review development and writing of the work. JL-V, JL, and JC: substantial contributions to the design of the work. MD-M, KM, JL, and JC: revised the work critically for important intellectual content. JC: final approval of the version to be published. All authors contributed to the article and approved the submitted version.

## Funding

This work was supported by the PI20/01471 project, integrated in the Plan National de I+D+I, AES 2020–2023; funded by the ISCIII and co-funded by the European Regional Development Fund (ERDF).

## Conflict of interest

The authors declare that the research was conducted in the absence of any commercial or financial relationships that could be construed as a potential conflict of interest. The reviewer VFR declared a shared affiliation with the authors KM, MD-M, AD, AG-M, JL, and JC to the handling editor at the time of review.

## Publisher's note

All claims expressed in this article are solely those of the authors and do not necessarily represent those of their affiliated organizations, or those of the publisher, the editors and the reviewers. Any product that may be evaluated in this article, or claim that may be made by its manufacturer, is not guaranteed or endorsed by the publisher.

## References

[1] American Psychiatric Association. (2013). Diagnostic and Statistical Manual of Mental Disorders, 5th Edn. Washington, DC: American Psychiatric Association. 10.1176/appi.books.9780890425596

[2] Thomas N Gurvich C Kulkarni J. Borderline personality disorder, trauma, and the hypothalamus–pituitary–adrenal axis. Neuropsychiatr Dis Treat. (2019) 15:2601–12. 10.2147/NDT.S198804 31564884 PMC6743631

[3] Gurvits IG Koenigsberg HW Siever LJ. Neurotransmitter dysfunction in patients with borderline personality disorder. Psychiatr Clin North America. (2000) 23:27–40. 10.1016/S0193-953X(05)70141-6 10729929

[4] Vogel F Wagner S Baskaya O Leuenberger B Mobascher A Dahmen N. Variable number of tandem repeat polymorphisms of the arginine vasopressin receptor 1A gene and impulsive aggression in patients with borderline personality disorder. Psychiatr Genet. (2012) 22:105–6. 10.1097/YPG.0b013e32834accad 22008661

[5] Coccaro EF Lee R Kavoussi RJ. Aggression, suicidality, and intermittent explosive disorder: serotonergic correlates in personality disorder and healthy control subjects. Neuropsychopharmacology. (2010) 35:435–44. 10.1038/npp.2009.148 19776731 PMC3055394

[6] Zimmerman DJ Choi-Kain LW. The hypothalamic-pituitary-adrenal axis in borderline personality disorder: a review. Harv Rev Psychiatry. (2009) 17:167–83. 10.1080/10673220902996734 19499417

[7] Carrasco JL Díaz-Marsá M Pastrana JI Molina R Brotons L López-Ibor MI. Hypothalamic-pituitary-adrenal axis response in borderline personality disorder without post-traumatic features. Br J Psychiat. (2007) 190:357–358. 10.1192/bjp.bp.106.022590 17401044

[8] Harned MS Rizvi SL Linehan M. Impact of co-occurring posttraumatic stress disorder on suicidal women with borderline personality disorder. Am J Psychiat. (2010) 167:1210–7. 10.1176/appi.ajp.2010.09081213 20810470

[9] Pietrzak RH Goldstein R Southwick SM Grant BF. Personality disorders associated with full and partial posttraumatic stress disorder in the US population: results from Wave 2 of the National Epidemiologic Survey on Alcohol and Related Conditions. J Psychiat Res. (2011) 45:678–86. 10.1016/j.jpsychires.2010.09.013 20950823 PMC3388551

[10] Kingdon DG Ashcroft K Bhandari B Gleeson S Warikoo N Symons M. Schizophrenia and borderline personality disorder: similarities and differences in the experience of auditory hallucinations, paranoia, and childhood trauma. J Nerv Ment Dis. (2010) 198:399–403. 10.1097/NMD.0b013e3181e08c27 20531117

[11] Tyrka AR Wyche MC Kelly MM Price LH Carpenter LL. Childhood maltreatment and adult personality disorder symptons: influence of maltreatment type. Psychiatry Res. (2009) 165:281–97. 10.1016/j.psychres.2007.10.017 19162332 PMC2671800

[12] Zanarini MC Frankenburg FR Hennen J Reich DB Silk KR. The McLean Study of Adult Development (MSAD): overview and implications of the first six years of prospective follow-up. J Pers Disord. (2005) 19:505–23. 10.1521/pedi.2005.19.5.505 16274279

[13] Sansone RA Gaither GA Songer DA. The relationships among childhood abuse, borderline personality, and self-harm behavior in psychiatric inpatients. Violence Vict. (2002) 17:49–55. 10.1891/vivi.17.1.49.33636 11991156

[14] Deckers JW Lobbestael J Van Wingen GA Kessels RP Arntz A Egger JI. The influence of stress on social cognition in patients with borderline personality disorder. Psychoneuroendocrinology. (2015) 52:119–129. 10.1016/j.psyneuen.2014.11.003 25459898

[15] Duesenberg M Wolf OT Metz S Roepke S Fleischer J Elias V. Psychophysiological stress response and memory in borderline personality disorder. Eur J Psychotraumatol. (2019) 10:1568134. 10.1080/20008198.2019.1568134 30788063 PMC6374976

[16] Inoue A Oshita H Maruyama Y Tanaka Y Ishitobi Y Kawano A. Gender determines cortisol and alpha-amylase responses to acute physical and psychosocial stress in patients with borderline personality disorder. Psychiatry Res. (2015) 228:46–52. 10.1016/j.psychres.2015.04.008 25979467

[17] Rinne T De Kloet ER Wouters L Goekoop JG DeRijk RH Van den Brink W. Hyperresponsiveness of hypothalamic-pituitary-adrenal axis to combined dexamethasone/corticotropin-releasing hormone challenge in female borderline personality disorder subjects with a history of sustained childhood abuse. Biol Psychiatry. (2002) 52:1102–12. 10.1016/S0006-3223(02)01395-1 12460693

[18] Wingenfeld K Spitzer C Rullkötter N Löwe B. Borderline personality disorder: hypothalamus pituitary adrenal axis and findings from neuroimaging studies. Psychoneuroendocrinology. (2010) 35:154–70. 10.1016/j.psyneuen.2009.09.014 19837517

[19] Timmermans S Souffriau J Libert CA. General introduction to glucocorticoid biology. Front Inmunol. (2019). 10:1545. 10.3389/fimmu.2019.01545 31333672 PMC6621919

[20] McNally JG Müller WG Walker D Wolford R Hager GL. The glucocorticoid receptor: rapid exchange with regulatory sites in living cells. Science. (2000) 287:1262–5. 10.1126/science.287.5456.1262 10678832

[21] Pace T Miller A. Cytokines and glucocorticoid receptor signaling. Relevance to major depression. Ann N Y Acad Sci. (2009) 1179:86–105. 10.1111/j.1749-6632.2009.04984.x 19906234 PMC3399249

[22] De Kloet CS Vermetten E Bikker A Meulman E Geuze E Kavelaars A. Leukocyte glucocorticoid receptor expression and immunoregulation in veterans with and without post-traumatic stress disorder. Mol Psychiatry. (2007) 12:443–53. 10.1038/sj.mp.4001934 17245326

[23] First MB Gibbon M Spitzer RL Williams JB Benjamin LS. Structured Clinical Interview for DSM-IV Axis II Personality Disorders, (SCID-II). Washington DC: American Psychiatric Press, Inc. (1997).

[24] Perez V Barrachina J Soler J Pascual JC Campins MJ Puigdemont D. The clinical global impression scale for borderline personality disorder patients (CGI-BPD): a scale sensible to detect changes. Actas Españolas de Psiquiatrí*a*. (2007) 35:229–35. 17592784

[25] Hall RC. Global assessment of functioning. A modified scale. Psychosomatics. (1995) 3:267–75. 10.1016/S0033-3182(95)71666-8 7638314

[26] Davidson JR Smith R. Traumatic experiences in psychiatric outpatients. J Trauma Stress. (1990) 3:459–75. 10.1002/jts.2490030314

[27] Hamilton M. The assessment of anxiety states by rating. Br Med J Psychol. (1959) 32:50–5. 10.1111/j.2044-8341.1959.tb00467.x 13638508

[28] Montgomery SA Åsberg MA. A new depression scale designed to be sensitive to change. Br J Psychiat. (1979) 134:382–389. 10.1192/bjp.134.4.382 444788

[29] Díaz-Marsá M MacDowell KS Guemes I Rubio V Carrasco JL Leza JC. Activation of the cholinergic anti-inflammatory system in peripheral blood mononuclear cells from patients with borderline personality disorder. J Psychiatr Res. (2012) 46:1610–7. 10.1016/j.jpsychires.2012.09.009 23083519

[30] García-Bueno B Bioque M MacDowell KS Barcones MF Martínez-Cengotitabengoa M Pina-Camacho L. Pro-/anti-inflammatory dysregulation in patients with first episode of psychosis: toward an integrative inflammatory hypothesis of schizophrenia. Schizophr Bull. (2014) 40:376–87. 10.1093/schbul/sbt001 23486748 PMC3932081

[31] Sabo AN. Etiological significance of associations between childhood trauma and borderline personality disorder: conceptual and clinical implications. J Personality Disorders. (1997) 11:50–70. 10.1521/pedi.1997.11.1.50 9113822

[32] Lorenzini N Fonagy P. Attachment and personality disorders: a short review. FOCUS: J Lifelong Learn Psychiat. (2013) 11:155–66. 10.1176/appi.focus.11.2.155

[33] Hooley JM Wilson-Murphy M. Adult attachment to transitional objects and borderline personality. J Pers Disord. (2012) 26:179–91. 10.1521/pedi.2012.26.2.179 22486448

[34] MacDowell KS Marsá MD Buenache E Villatoro JM Moreno B Leza JC. Inflammatory and antioxidant pathway dysfunction in borderline personality disorder. Psychiatry Res. (2020) 284:112782. 10.1016/j.psychres.2020.112782 31955054

[35] García-Bueno B Caso JR Leza JC. Stress as a neuroinflammatory condition in brain: damaging and protective mechanisms. Neurosci Biobehav Rev. (2008) 32:1136–51. 10.1016/j.neubiorev.2008.04.001 18468686

